# Sequential Membrane Rupture and Vesiculation during *Plasmodium berghei* Gametocyte Egress from the Red Blood Cell

**DOI:** 10.1038/s41598-018-21801-3

**Published:** 2018-02-23

**Authors:** Maria Andreadaki, Eric Hanssen, Elena Deligianni, Cyrille Claudet, Kai Wengelnik, Vanessa Mollard, Geoffrey I. McFadden, Manouk Abkarian, Catherine Braun-Breton, Inga Siden-Kiamos

**Affiliations:** 1 Institute of Molecular Biology and Biotechnology, Foundation for Research and Technology - Hellas, Heraklion, Greece; 2 Department of Biology, University of Crete, Heraklion, Greece; 3 Bio21 Molecular Science and Biotechnology Institute, Electron Microscopy Unit, University of Melbourne, Melbourne, Victoria, Australia; 4 Department of Biochemistry and Molecular Biology, University of Melbourne, Melbourne, Victoria Australia; 5 Université de Montpellier, CNRS UMR 5048, INSERM UMR 1054 Montpellier, France; 6 Université de Montpellier, CNRS UMR 5235 Montpellier Cedex, France; 7 School of BioSciences, University of Melbourne, Melbourne, Victoria, Australia

## Abstract

Malaria parasites alternate between intracellular and extracellular stages and successful egress from the host cell is crucial for continuation of the life cycle. We investigated egress of *Plasmodium berghei* gametocytes, an essential process taking place within a few minutes after uptake of a blood meal by the mosquito. Egress entails the rupture of two membranes surrounding the parasite: the parasitophorous vacuole membrane (PVM), and the red blood cell membrane (RBCM). High-speed video microscopy of 56 events revealed that egress in both genders comprises four well-defined phases, although each event is slightly different. The first phase is swelling of the host cell, followed by rupture and immediate vesiculation of the PVM. These vesicles are extruded through a single stabilized pore of the RBCM, and the latter is subsequently vesiculated releasing the free gametes. The time from PVM vesiculation to completion of egress varies between events. These observations were supported by immunofluorescence microscopy using antibodies against proteins of the RBCM and PVM. The combined results reveal dynamic re-organization of the membranes and the cortical cytoskeleton of the erythrocyte during egress.

## Introduction

Egress from the host cell is essential for the completion of the life cycle of intracellular pathogens. *Plasmodium* parasites, alternating between intracellular stages and extracellular invasive stages, must exit host cells during key transition stages. One of these is gametogenesis, which takes place during transmission to the mosquito. During a brief interval, male and female gametocytes, having developed inside the parasitophorous vacuole (PV) in the red blood cell (RBC), are released. Microscopy studies suggest that gametocyte egress is similar to merozoite egress in that the PV membrane (PVM) ruptures first and the RBC membrane (RBCM) opens later, which is consistent with the so called inside-out model of egress^1,2^.

The PVM is formed during invasion of the red blood cell and is believed to contain lipids originating both from the host cell and secreted from the invading parasite (for a review see^3^). A limited number of proteins of parasite origin are residents in the PVM, while proteins of the host cell are mostly excluded. Some PVM proteins are shared between gametocytes and the asexual blood stages, such as subunits of the translocon^4^, but there are also important differences. One example is the different members of the ETRAMP protein family^3^. In *P. falciparum* the ETRAMP family protein *Pf*s16 is specifically present in the gametocyte PVM^5,6^ and gene disruption studies suggest that it is important for successful development of gametocytes^7^.

Egress has been studied by reverse-genetic approaches, and several proteins are confirmed to have roles in egress. Most studies have been performed in *P. berghei*. For instance, MDV/PEG3^8^ and PbGEST^9^ function in the rupture of the PVM in both genders, and are localized to specialized secretory organelles called osmiophilic bodies. Mutant male gametocytes lacking the stage-specific actin isoform, actin 2, are also unable to rupture the PVM, but egress of the female gametocytes is not affected in this mutant^10^. In these three mutants both surrounding membranes remain intact. Another mutant lacking PPLP2, one of the five perforin-like proteins, in *P. berghei*, also has a male-specific phenotype. However, in this disruption mutant of *pplp2* the PVM ruptures normally while the RBCM remains intact^1^. In many mutant cells lacking PPLP2 the cytoplasm of the red blood cell is also visible in EM pictures (unpublished^1^). A female-specific protein called G377 also has a minor role in egress^11^. Inhibitor studies suggest that proteases have important roles during egress, but no protease has so far been identified with a role in this process^12^. Recently a proteomics approach identified a TRAP-like protein, called MTRAP, residing in osmiophilic bodies. In its absence gametes were trapped in the host cell^13^. Another study confirmed this role of MTRAP in both *P. berghei* and *P. falciparum* gametogenesis and also showed that in disruption mutants neither the PVM nor the RBCM were ruptured. Furthermore, the protein was important for egress of both genders^14^.

Gametocytogenesis is initiated when merozoites already committed to sexual development through the activity of the transcription factor ApiAP2-G, invade RBCs^15,16^. The early stages of the developing gametocytes are molecularly and morphologically similar to the asexual stages; they develop within the PVM, digest hemoglobin and modify the host red blood cell, but in later stages gametocytes follow a distinct developmental program^17^. After uptake of the mature gametocytes in a blood meal by the mosquito, gametogenesis is activated by the decrease in temperature and by xanthurenic acid (XA)^18^. XA triggers activation of cGMP dependent protein kinase (PKG)^19^ which mediates an increase in intracellular Ca^2+^ levels^20^ and hydrolysis of PIP_2_ and the production of IP_3_^21^ thus initiating egress of both genders. Effector proteins important for membrane rupture reside in osmiophilic bodies and their release is dependent upon intracellular Ca^2+^ levels^11^. The male gametocyte also undergoes three mitotic divisions, and assembly and activation of eight axonemes leads to the formation of eight motile flagellar gametes from each gametocyte. The females are believed to be competent for fertilization without any re-organization of the cell. Gametogenesis is completed 10–15 min after uptake of the blood by a mosquito.

In the asexual blood cycle invasive merozoites are released after initial rupture of the PVM and then subsequent opening of the RBCM^22^. Merozoite egress is also dependent on PKG activity^22,23^, elevated Ca^2+^ levels^24–26^ and PIP_2_ hydrolysis^20^. Parasite-derived proteases have important roles^27^ and host factors are necessary for disrupting the host cytoskeleton, a crucial step for RBCM rupture^24,28,29^. Detailed morphological studies by live imaging of *P. falciparum* merozoite egress revealed that a single stabilized pore in the RBCM is followed by outward curling and buckling of the membrane^30^. A similar outward curling has also been detected in egressing *P. falciparum* gametocytes^31^. An ultrastructural study revealed that egress is preceded by the loss of mechanical integrity of the RBC cytoskeleton, and at the same time the PVM ruptures into multilamellar vesicles. Next the RBCM becomes permeable followed by the explosive egress of the merozoites^22^. In the study presented here we use the term vesicles of the ruptured membranes, in line with that publication^22^.

Here, we investigated *P. berghei* gametocyte egress combining high speed video observations with immunofluorescence analysis using antibodies against the RBCM and PVM. Although each event differed, four main phases were identified: swelling; PVM rupture and vesiculation; a single pore opening in the RBCM; and, finally, rupture and vesiculation of the RBCM. The sequence of phases was similar between the two genders.

## Results

### High speed video recordings of gametocyte egress reveal rupture and vesiculation of the two surrounding membranes

**Figure 1:**
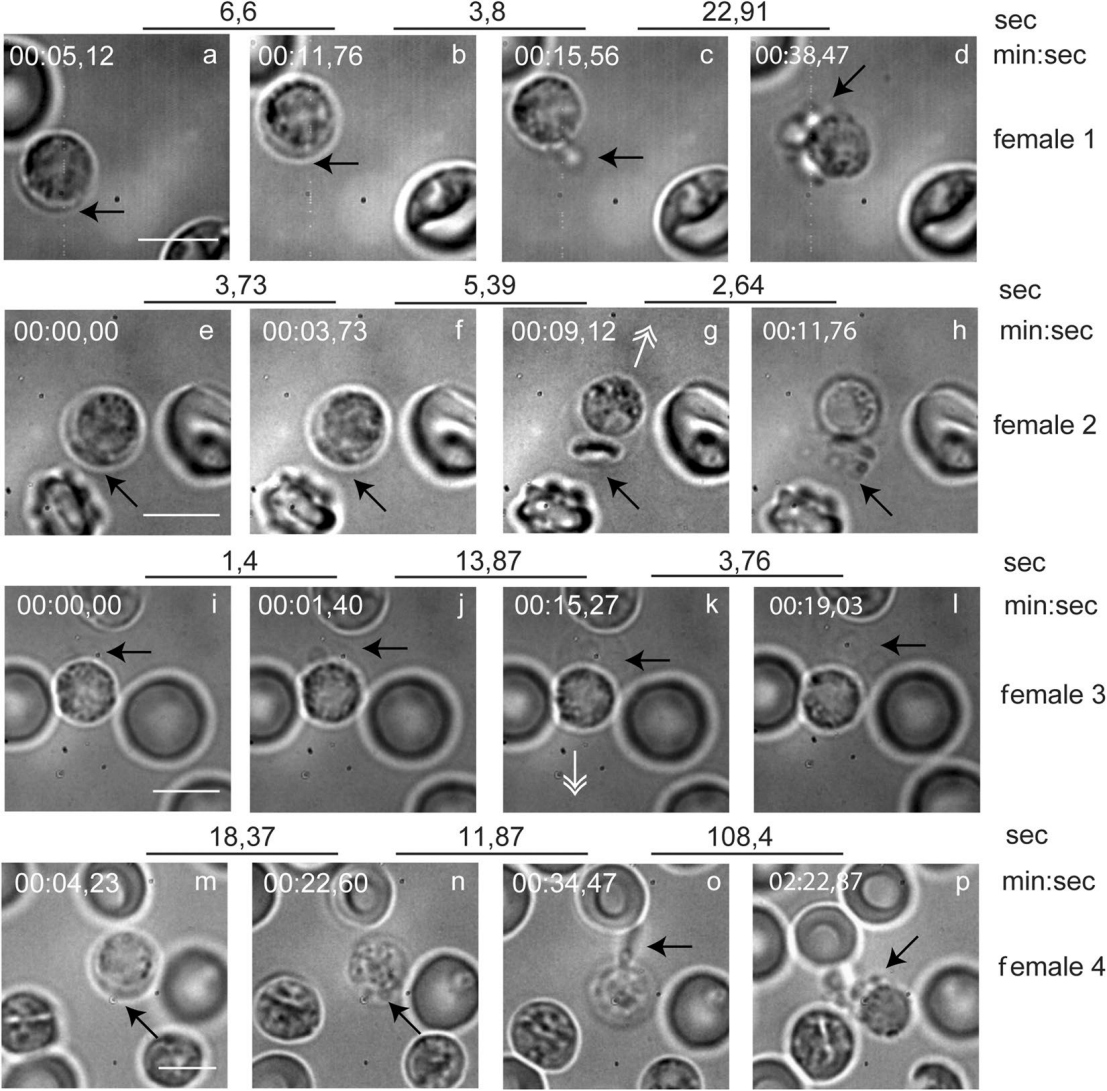
Snapshots from four movies of female gamete egress. Representative snapshots chosen to illustrate the stages of egress. Upper broken lines represent time in sec between each frame of the movies in real time. The time of each frame from the start of the movies is indicated above the snapshots (min:sec). For technical reasons this does not correspond with the time from the start of the experiment (activation). (**a**–**d**) Female 1. (**a**) An infected RBC in the initial phase of swelling. The parasite occupies most of the volume of the RBC. (**b**) The RBCM is more extended. (**c**) The PVM vesicles are extruded through a single pore. (**d**) Egress is complete. Vesiculated PVM and RBCM are localized in proximity to the gamete. (**e**–**h**) Female 2. (**e**,**f**) The PVM is already vesiculated inside the RBCM. (**g**) The RBCM is extended and the gamete slides out of the host membrane. (**h**) Egress is complete with vesicles next to the gamete. (**i–l**) Female 3. (**i**) The RBCM is largely extended. (**j**) PVM is ruptured and vesiculated. (**k**) The gamete slides out of the extended RBCM. (**l**) egress is complete, the RBCM is vesiculated. (**m**–**p**) Female 4. (**m**) RBC is swollen. (**n**) PVM vesicles are seen inside the RBCM towards the viewer. (**o**) Expulsion of PVM vesicles through a single pore. (**p**) Egress is complete. Black arrows indicate the area of interest in each snapshot, and white double arrows direction of gamete expulsion in (**g**) and (**k**). Scale bar, 5 μm. See also Supplementary Information Videos S1–S4.

**Figure 2:**
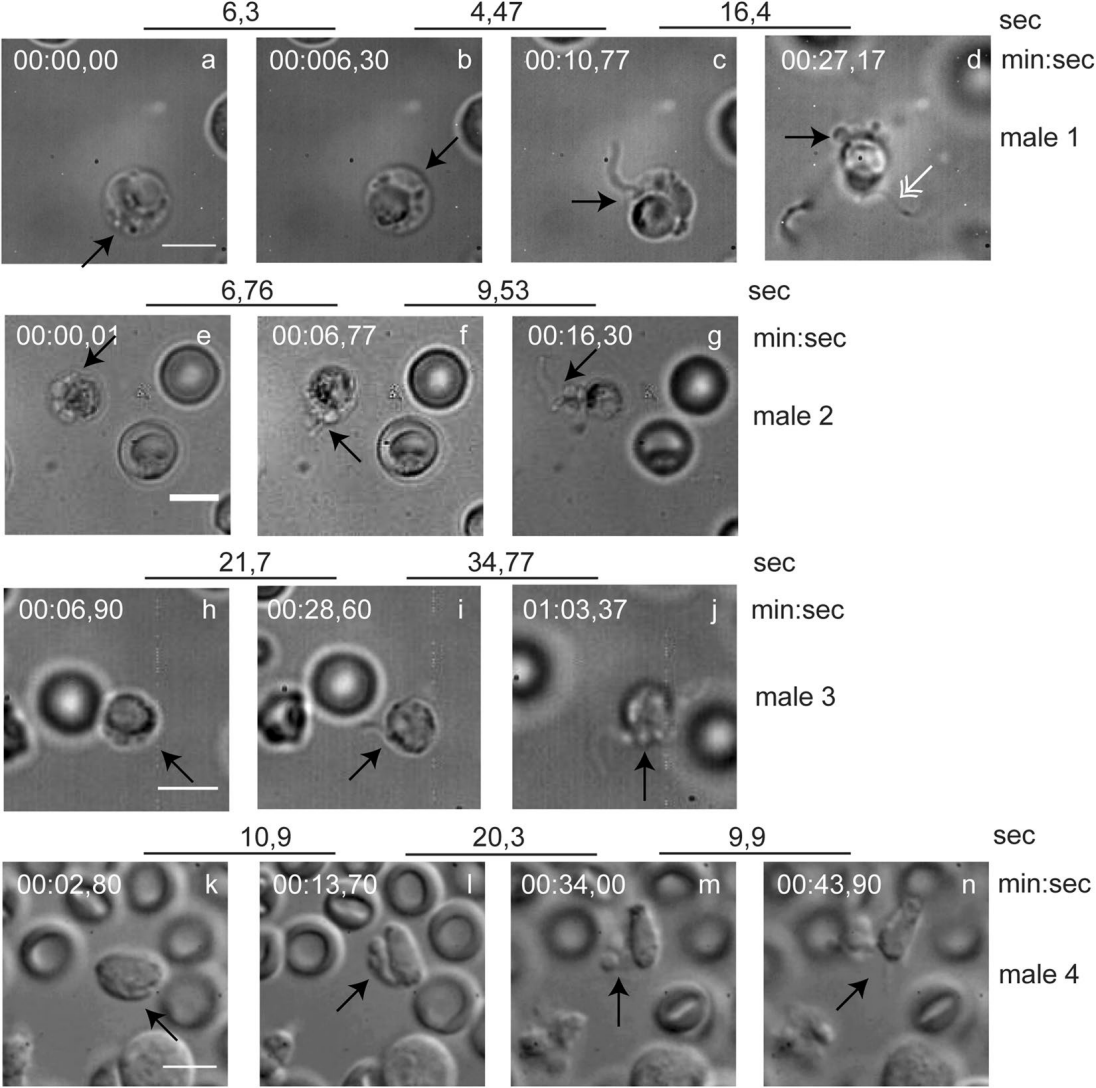
Snapshots from movies of four male gametocytes undergoing egress and exflagellation. Representative snapshots chosen to illustrate the stages of egress. Upper broken lines represent time in sec between each frame of the movies in real time. The time of each frame from the start of the movies is indicated above the snapshots (min:sec). For technical reasons this does not correspond with the time from the start of the experiment (activation). (**a**–**d**) Male 1. (**a**,**b**) The RBC is swollen with PVM vesicles visible. (**c**) A flagellum is exiting the RBCM through a single pore. (**d**) Egress is complete. Vesiculated PVM and RBCM are localized in proximity residual gametocyte and a free flagellum is also visible (white double arrow). (**e**–**g**) Male 2. (**e**) The PVM is already vesiculated inside the RBCM. (**f**) A vesicle exits the RBCM through a single pore followed by a single flagellum. (**g**) Egress is complete with vesicles and a free flagellum next to the residual gametocyte. (**h**–**j**) Male 3. (**h**) The RBCM is extended with PVM vesicles inside. (**i**) A single flagellum is exiting the RBCM through a single pore. (**j**) Egress is complete, with vesicles and one flagellum in proximity to the residual gametocytes. (**k**–**n**) Male 4. (**k**) The RBCM is extended. (**l**) The PVM is vesiculated. (**m**) Vesicles of RBCM and PVM are seen close to the cell. (**n**) Exflagellation complete. Black arrows indicate the area of interest in each snapshot. Scale bar, 5 μm. See also Supplementary Information Videos S5–S9.

We used a high-speed video camera to follow egress in real time. Blood samples from infected mice were diluted in medium, transferred to microscope slides and videos were recorded, beginning a few minutes after activation. Due to the short interval between activation and the first signs of egress the beginning was not always recorded. 56 events, of which 34 were females and 22 males, were captured. Four representative movies of each gender are presented (Snapshots Figs 1 and 2 and Supplementary Information Video 1–8). Egress does not follow a stereotyped pattern, but despite this, the series of events have common features. The first discernible feature is swelling of the erythrocyte (Figs 1b,m and 2k) with the parasite asymmetrically positioned in the host cell. Then the PVM ruptures and forms vesicles internal to the erythrocyte membrane (Figs 1e,f,i,j,n and 2a,b,e,h,l). The vesicles are freely moving within the erythrocyte, suggesting that the RBC cytoplasm is largely digested or has leaked out of the cell. Next, the erythrocyte membrane opens at a single pore and in the females the internal vesicles are discharged (Fig. 1c,o) or the gamete is released with the vesicles positioned on the opposite side of the pore (Fig. 1g,k). In the males the vesicles are accompanied by the exit of a single motile male flagellum (Fig. 2c,i), but in two cases we detected vesicle extrusion slightly earlier than activation of the flagella (Fig. 2f,m, Supplementary Information Video S6, S8). The pore is then widened with the expulsion of the gamete followed by destabilization of the pore’s rim, buckling of the RBCM and its shredding into small vesicles. The vesicles remain in the vicinity of the male residual gametocyte or female gamete for several minutes (Figs 1d,h,l,p and 2d,g,j,n).

We recorded the approximate timing of the different phases. In total we were able to time at least some of them for 10 females and 9 males. In only two events did we detect the beginning of the swelling and the PVM in these cases ruptured 66 sec and 375 sec after swelling. The interval between PVM rupture and pore opening varies from a few seconds to five minutes, whilst the RBCM vesiculation in eight cases happens within seconds but in two cases ~ 2 minutes after the opening (Supplementary Information Fig. S1a). Thus the intervals between the phases are highly variable, and this is the case for both genders.

To quantify the swelling that we observed we measured the area of the RBC and gametocyte in Giemsa stained smears of non-activated and activated gametocytes (Supplementary Information Fig. S1b). The ratio of the two values was plotted, and the difference comparing non-activated and activated cells was significant. The results are consistent with the live imaging although we cannot exclude that weakening of the host cell membrane after activation may make the cells more sensitive to the smearing on the slide, thus overestimating swelling.

### Live immunolabeling confirms single pore opening in the erythrocyte membrane and vesiculation of the membrane

**Figure 3:**
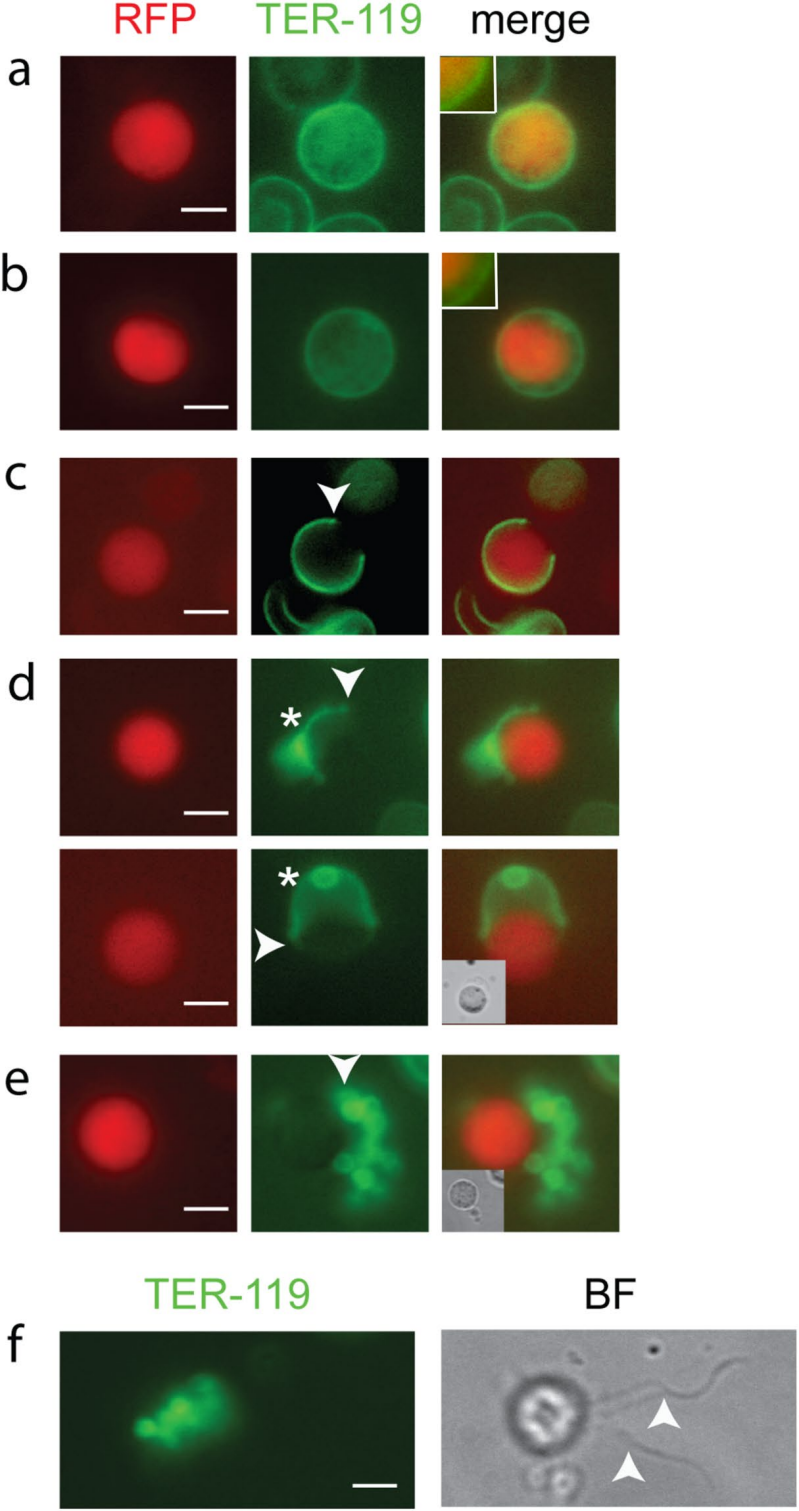
Live imaging reveals RBCM rupture. Samples were imaged without prior fixation. (**a**–**e**) Female cells expressing RFP (red) and labeled with TER-119 (green). (**a**) A female gametocyte still enclosed in the intact RBCM. The membrane is adjacent to the parasites. (**b**) Swelling of the RBCM membrane is evident. Insets show the adjacent (**a**) and displaced RBCM (**b**) compared to the parasite. (**c**) RBCM opened at a single point. Arrow points to a slight thickening of the membrane at the opening. (**d**) Two cells in the process of egress. The arrows point to a slight thickening of the membrane at the opening. Top panel: The RBCM is opening and simultaneously forming vesicles (asterisks, out of focus), the most common RBCM rupture detected. Lower panel: The RBCM opens as an intact membrane. Compare Fig. 1, female 2 and 3 and Supplementary Information Videos S2, S3. (**e**) Rupture of RBCM completed, and RBCM vesicles remain next to the gamete (white arrow). White arrow heads in (**c**–**e**) point to opened or vesiculated RBCM. (**f**) Egressed male which is exflagellating. RBCM vesicles remain in proximity to the residual gametocyte and two flagellar gametes are visible (BF image, white arrows). The cells are motile and thus slightly differently located between panels. Scale bar, 5 µm.

We used the TER-119 monoclonal antibody recognizing a glycophorin-A associated protein on the surface of the red blood cell to label the erythrocyte membrane. Blood samples were diluted in medium containing the antibody, and the cells were imaged in an epifluorescence microscope with an attached CCD camera. We used the *P. berghei* strain 820 in which female gametocytes express red fluorescent protein (RFP), while the males express a weak green fluorescent protein (GFP) not detectable in these experiments^8^. The flagellar male gametes move rapidly, precluding good quality live imaging. Therefore, we focused on female cells. Female gametocytes were observed that were enclosed in the RBCM being adjacent to the parasite (Fig. 3a). In other cells swelling of the host cell was seen as the RBCM was now separate from the parasite (Fig. 3b). The actual opening of the pore was not captured, possibly due to the very brief time before the wider opening of the pore. However, we did detect a partially opened membrane (Fig. 3c). Typically, the final opening of the membrane is accompanied by vesiculation of the membrane (Fig. 3d, upper panel). However, we also detected a gamete having almost exited the intact membrane (Fig. 3d, lower panel, compare to Fig. 1g,k and Supplementary Information Video S2,S3). We did not unequivocally recognize the characteristic curling of the membrane, previously described for egress of *P. falciparum* merozoites^30^ and gametocyte^31^, although in Fig. 3c and d there is a slight thickening of the membrane at the opening. However, the observed buckling of the RBC membrane following female gamete expulsion is indicative of curling. This is most clearly visible in Supplementary Information Video S2.

After completion of gametogenesis we detected labeled membrane vesicles in the proximity of the female gamete (Fig. 3e) and the residual gametocyte of exflagellating cells despite the vigorous beating of the flagellar gametes (Fig. 3f). These observations confirm that the RBCM is vesiculated after egress and that the vesicles remain close to the cells in both genders.

### Outside-out vesiculation of the red cell membrane

**Figure 4:**
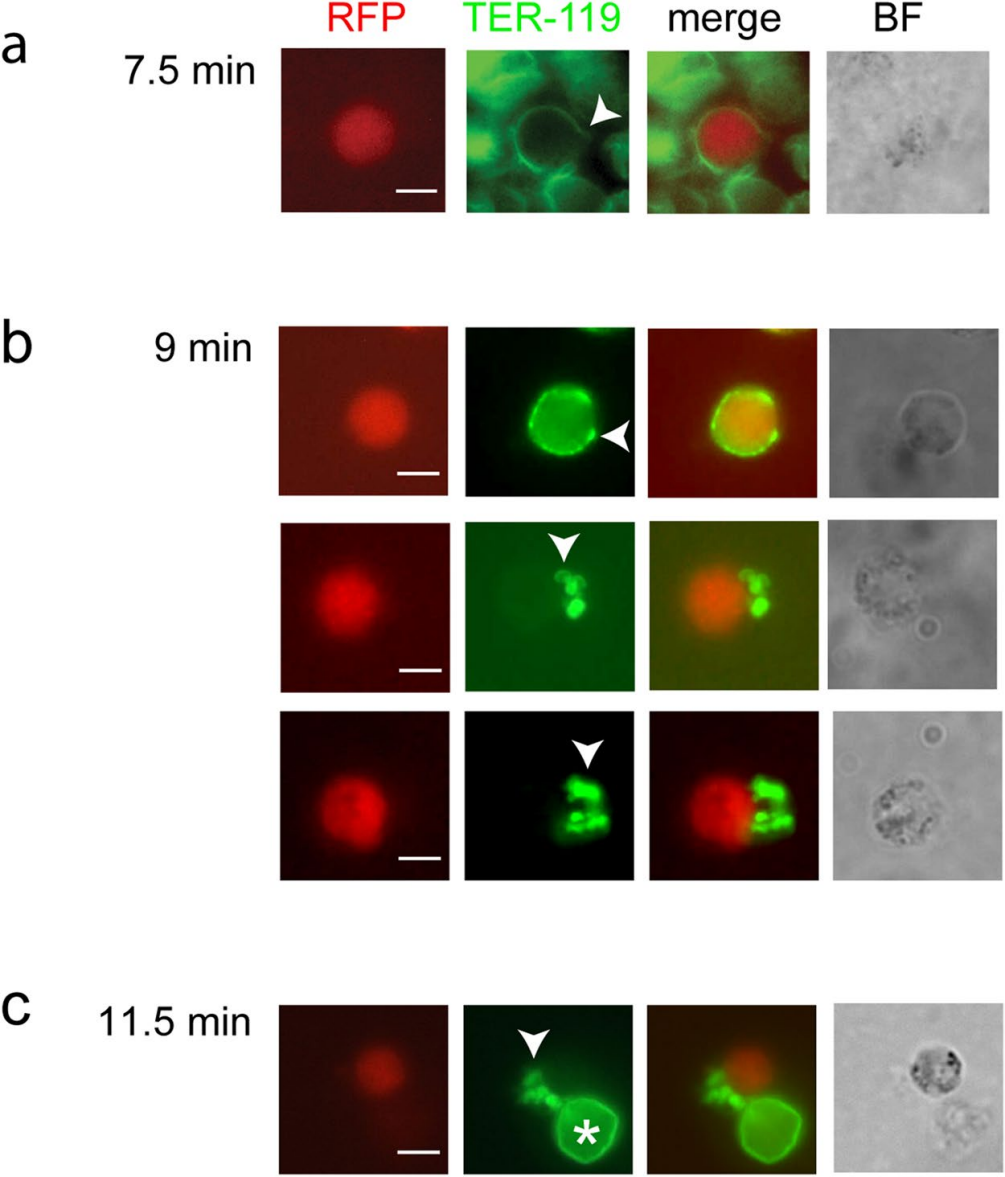
Outside-out vesiculation revealed with TER-119 label. (**a**–**c**) Females (expressing RFP, red) were fixed at different time points after activation and the RBCM labeled with TER-119 (green). (**a**) 7.5 min after activation the RBCM has opened at a single site. (**b**) Three females having completed gametogenesis 9 min after activation. Labeled outside-out vesicles remain in proximity to the gametes. (**c**) Completed gametogenesis at 11.5 min. Labeled vesicles remain close to the gamete. An intact RBC is indicated with an asterisk. White arrows point to opened or vesiculated RBCM. Scale bar, 5 µm.

We next performed immunofluorescence assays using the TER-119 antibody to label specifically the outer leaflet of the RBCM. Strain 820 gametocytes were fixed in formaldehyde without permeabilization at different time points after activation. We were not able to visualize the membrane just after opening of the pore, which is not unexpected given that our live imaging shows this to be a very rapid event. However, one example is shown where the membrane has opened but not yet vesiculated (Fig. 4a). We readily detected cells associated with free TER-119 labeled vesicles (Fig. 4b,c), confirming the live imaging results and indicating that the vesicles are of the outside-out type, that is, the outer leaflet of the membrane remains on the outside of the vesicles. However, we cannot exclude that inside-out vesicles are also formed but we were unable to test this possibility due to the lack of a marker for the inner leaflet of the RBCM.

### PVM visualized by SEP1 immunolabeling

**Figure 5:**
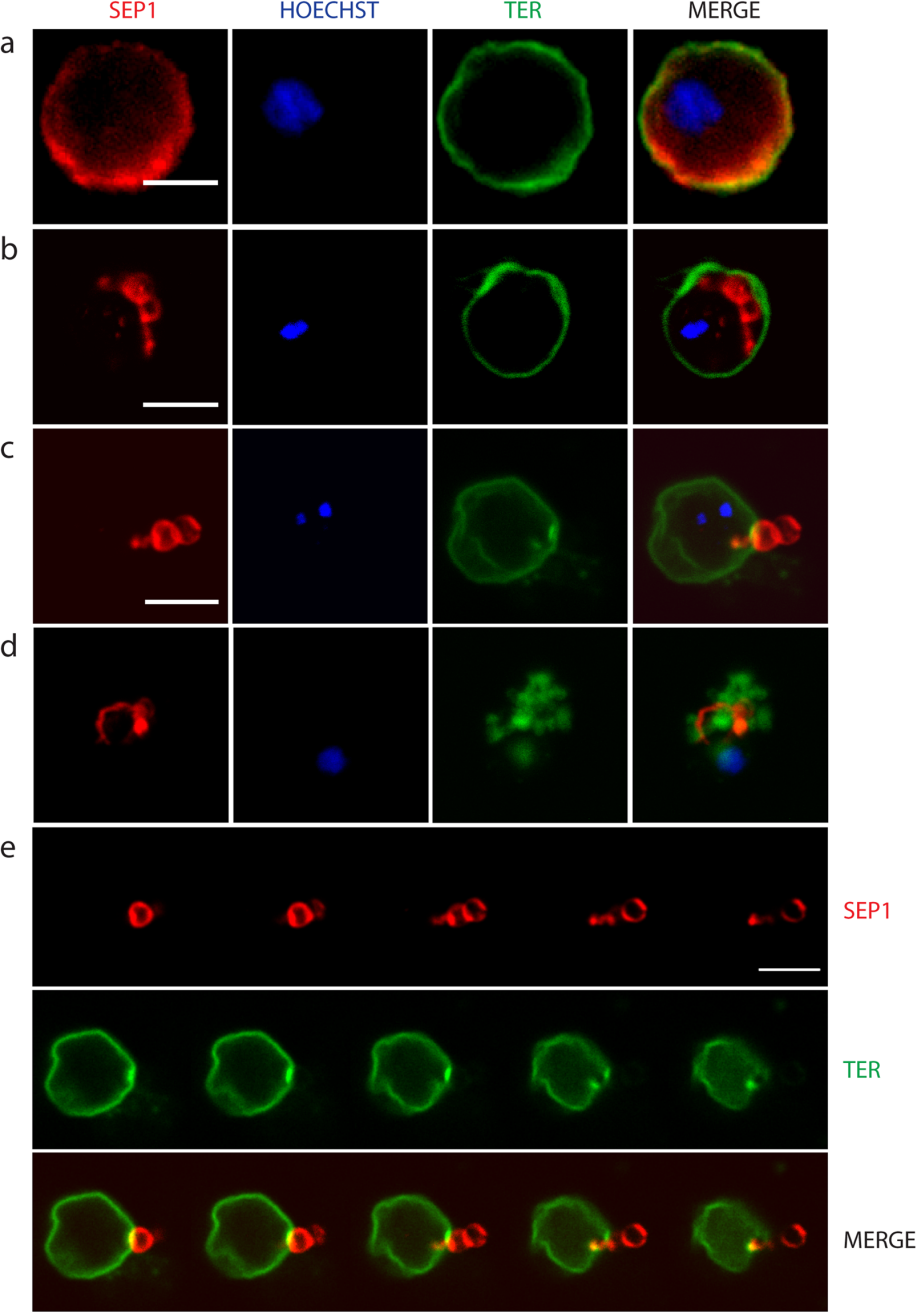
Labeling with SEP1 antibodies reveal PVM vesicles. Cells were labeled with the SEP1 antibody (red) and TER-119 (green) highlighting the PVM and RBCM, respectively. DNA is stained with Hoechst 33342 (blue). (**a**–**d**) Are projections of sections from confocal imaging. (**a**) Non-activated cell. (**b**) An activated cell. PVM vesicles have formed inside the intact RBCM. (**c**) The RBCM is opened at a single pore and SEP1 labeled vesicles are in the process of exiting. A montage of the same cell is shown in e. (**d**) A cell where egress is completed. Vesicles of RBCM and PVM are intermingled. (**e**) Montage of single sections of the cell in (**c**). Scale bar, 5 µm.

We used an antibody directed against the SEP1 protein, a member of the ETRAMP (early transcribed membrane proteins) protein family, previously shown to be located in the PVM, to visualize directly the PVM^32^. The immunolabelling of SEP1 revealed the closed PVM in non-activated samples (Fig. 5a) and SEP1 labeled vesicles inside intact RBCM in activated gametocytes (Fig. 5b). We also captured labeled vesicles in the process of exiting the RBCM (Fig. 5c,e). This confirms the results from the live imaging that PVM vesicles are extruded through a single pore opening of the RBCM. In addition a thickening of the RBCM around the pore was seen suggesting curling of the membrane. In another cell the PVM was seen close to the vesiculated RBCM (Fig. 5d).

**Figure 6:**
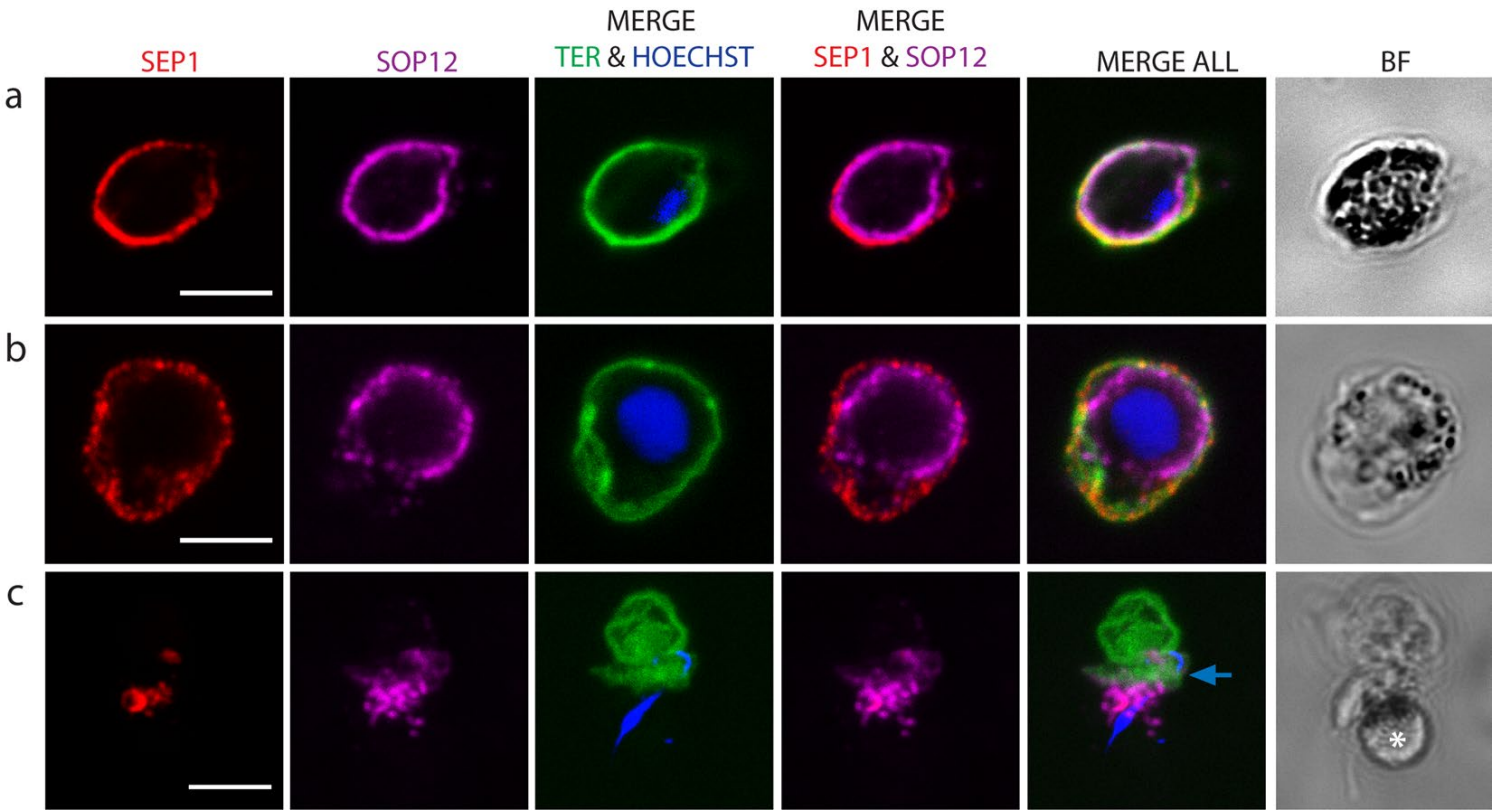
Labeling of SOP12-EGFP gametocytes reveal swelling of PV. The PVM was labeled with the SEP1 antibody (red), the parasite membrane protein SOP12 is highlighted with a GFP antibody (magenta) and the RBCM labeled with TER-119 (green). DNA is stained with Hoechst 33342 (blue). (**a**) Non-activated gametocyte. SOP12 is localized adjacent to the PVM and RBCM. (**b**) Activated gametocyte. The RBCM and PVM are extended, indicating the swelling of the PV and shrinking of the RBC cytoplasm. SOP12 is delineating the gametocyte. (**c**) Exflagellating male. The SOP12 label is no longer clearly delineated suggesting that the protein is secreted. Blue arrow shows the RBC membrane vesicles and above them there is another intact RBC. White asterisk shows the parasite. Projections of confocal stacks through the middle of the cell in a and b, and through the whole cell in c. Scale bar, 5 µm.

To visualize the gametocyte during egress we used PSOP12 as a marker; this protein has been shown to be located on the gametocyte surface^33^. Gametocytes of a strain expressing PbSOP12 fused to GFP^33^ were labeled with the SEP1 and TER-119 antibodies as above. In non-activated cells the signals of the three proteins were largely overlapping (Fig. 6a). In a cell where swelling had taken place SEP1 and TER-119 were detected adjacent to each other, while the SOP12 labeled the gametocyte inside the extended PVM (Fig. 6b). However, in exflagellating gametocytes we failed to detect PSOP12 circumferential to the gametocyte/gamete. Instead staining was seen in the vicinity of the cell suggesting that the protein is secreted after activation and trapped between the ruptured PVM and RBC membranes (Fig. 6c). Hence it was not possible to use this marker for labeling the egressing gamete.

### The PVM is vesicularized in parasites lacking Perforin-like protein 2

**Figure 7:**
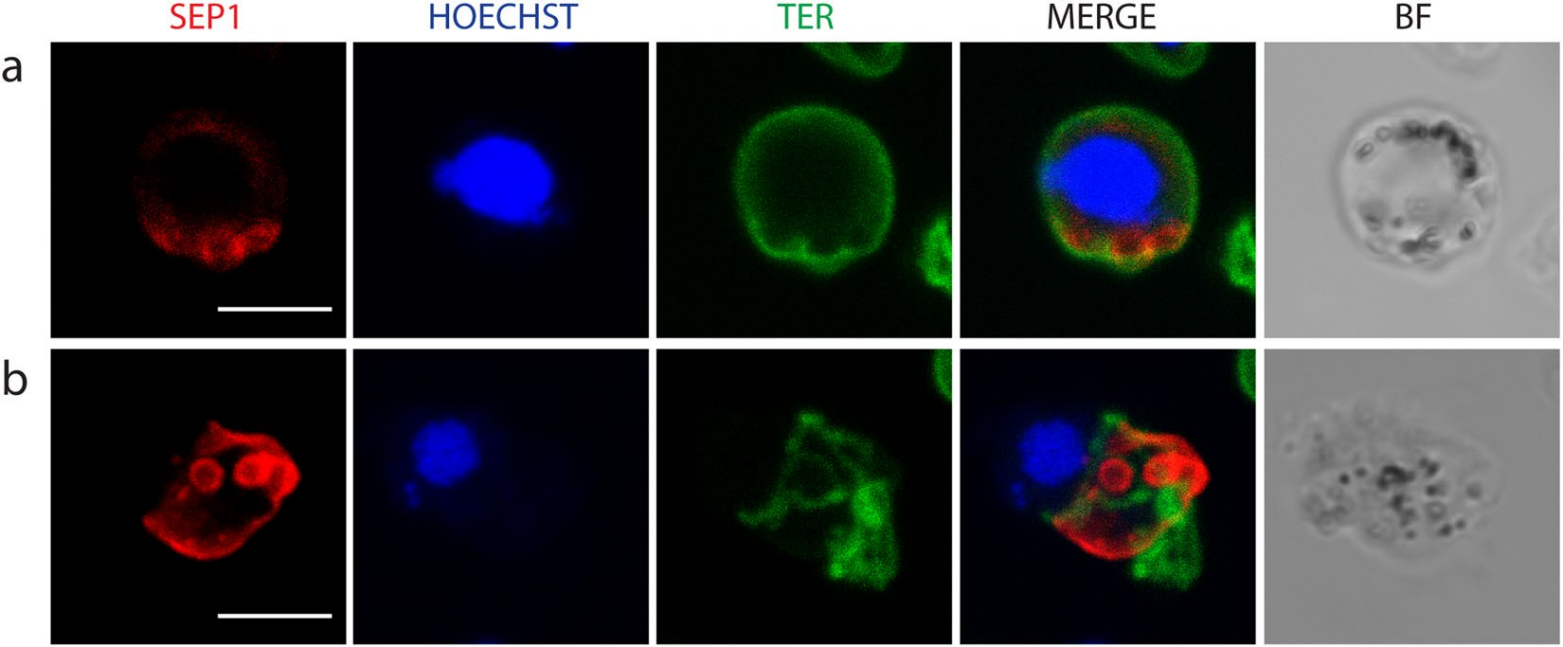
Parasites lacking PPLP2 form PVM vesicles. Activated *pplp2(-)* gametocytes labeled with the SEP1 antibody (red) and TER-119 (green). DNA is stained with Hoechst 33342 (blue). The samples were treated with equinatoxin which destabilizes the RBCM. (**a**) SEP1 labeled vesicles are formed inside the intact RBCM. (**b**) SEP1 labeled vesicles close to a partially ruptured RBCM.

We reported previously that in mutant male gametocytes lacking *P. berghei* perforin-like protein 2 (PPLP2) the PVM ruptured during activation of gametogenesis while the RBCM remained intact^1^. However, in that study we were not able to image the PVM. To confirm the earlier study and verify that the rupture of PVM is independent of that of RBCM we imaged activated *pplp2(−)* mutant gametocytes. As expected, PVM vesicles were formed inside the RBC membrane (Fig. 7a). In these experiments equinatoxin was used to permeabilize the RBC for efficient antibody labeling and this led in certain cases to collapse of the RBCM and release of PVM vesicles (Fig. 7b). In this case we did not detect the typical RBCM vesicles.

### Electron microscopy analysis of egressing parasites

**Figure 8:**
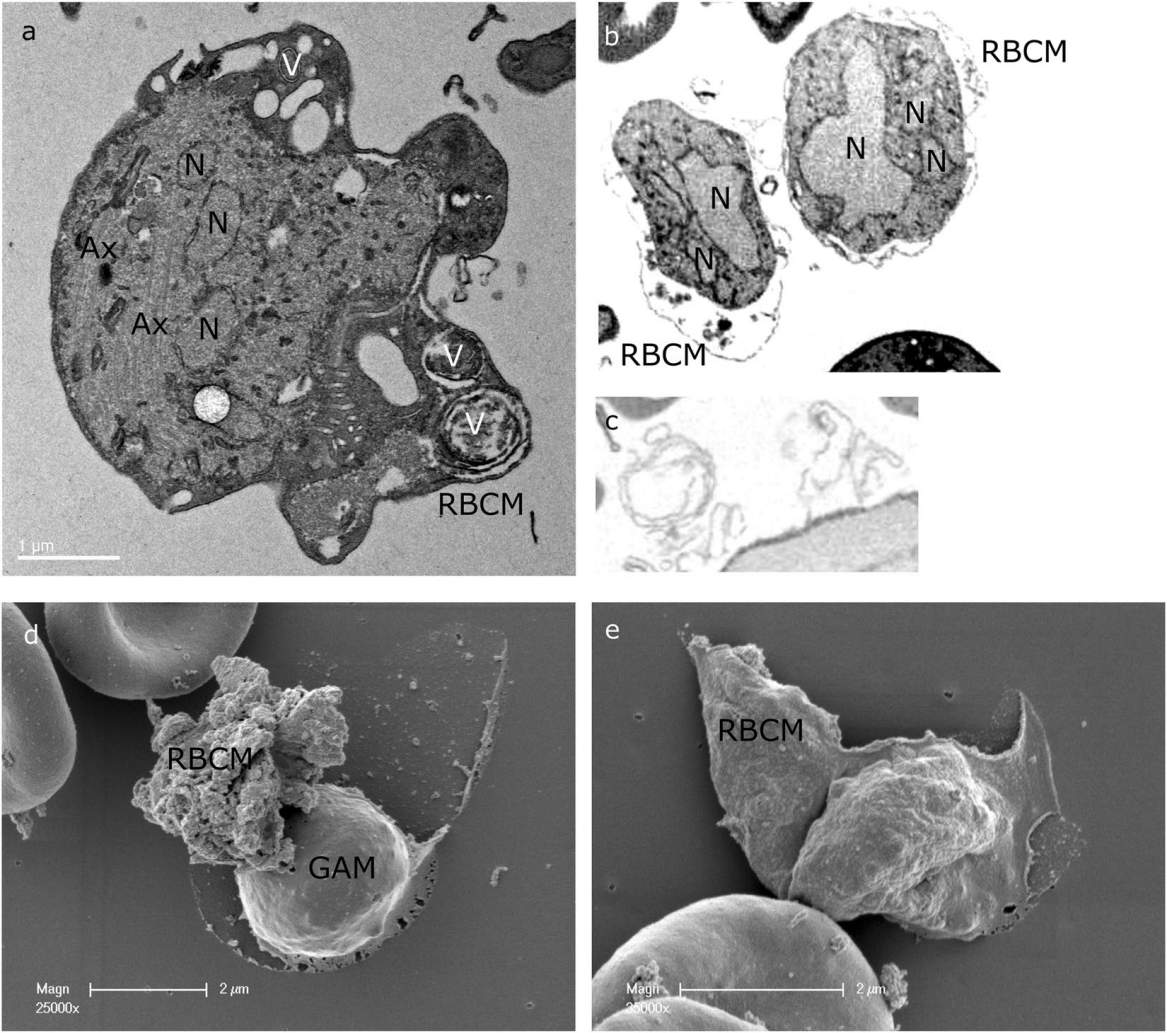
Electron microscopy analysis. (**a**) An activated male gametocyte. Inside the intact RBCM three vesicles are visible (V) probably derived from the PVM. No PVM surrounds the gametocyte. (**b**) Two activated gametocytes where the red blood cell cytoplasm has been completely digested. The RBCM is still intact. (**c**) Membrane spirals and whorls detected free in samples of activated gametocytes. (**d,e**) Scanning EM of activated gametocytes. (**d**) A cell is surrounded by a thin membrane collapsed on the grid. A convoluted empty membrane is located next to it. The rough surface suggests that the inner leaflet faces outwards. (**e**) An intact membrane sheet collapsed on a cell suggesting that the membrane was opened at one point and the opening then extended. The membrane is smooth contrasting to d. Ax, axoneme; N, nucleus; RBCM, red blood cell membrane; GAM, gamete.

Our light microscopy analysis was supported by electron microscopy of activated gametocytes. An example of a gametocyte still enclosed in the intact RBCM but with no visible PVM is presented (Fig. 8a); three membrane vesicles, one of which is flattened, are seen in the space outside the parasite. We interpret this as vesicles derived from the PVM consistent with the live imaging experiments and the SEP1 localization. In other cells the RBC cytoplasm was no longer visible, showing the gametocytes surrounded by the RBCM (Fig. 8b). We also detected free membrane whorls and spirals (Fig. 8c); it is not however possible to determine whether they are derived from the PVM or the RBCM. A scanning EM (SEM) analysis of samples from a time point when exflagellation took place revealed ruptured membranes (Fig. 8d,e), possibly derived from the RBCM. In one case (Fig. 8d) the membrane has a rough surface which can be interpreted to be the inner leaflet of the RBCM. Another membrane collapsed over a cell has a smoother surface suggesting the outer leaflet in this case facing outwards (Fig. 8e). This membrane is visible as one sheet further strengthening the view that the membrane is opened at a single pore which is then extended before vesiculation takes place.

## Discussion

**Figure 9:**
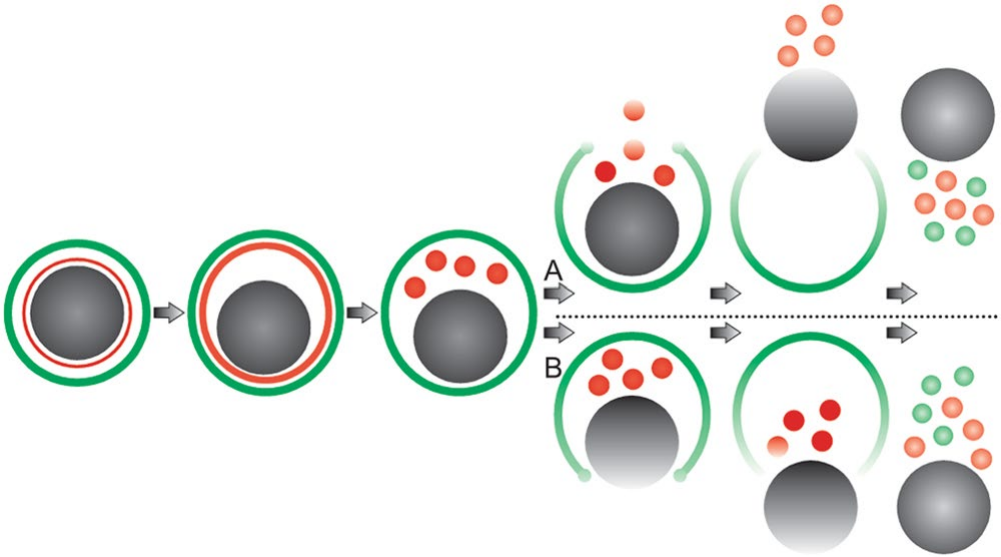
New model for egress. The gametocyte (gray) is surrounded by the PVM (red) and RBCM (green). The first sign of egress is swelling of the two membranes. Next, rupture and vesiculation of the PVM take place followed by the opening of a single stabilized pore in the RBCM. Two alternative positions of the pore were seen. The most common case was localization of the pore towards the PVM vesicles (A), which was seen in most females and all males. In more rare cases, and only detected in females, the pore was sited towards the parasite (B). Curling of the pore was suggested by the thickening of the rim detected in some events. The last step is the vesiculation of the RBCM resulting in a free female gamete or male residual cell.

Malaria parasites use unique processes to escape from the red blood cell. In this study we applied complementary imaging methods for a detailed analysis of *P. berghei* gametocyte egress from the host cell. The process does not follow a stereotyped pattern as each event that we observed was different and the timing of events also varies. However, egress can be divided into discreet steps which are similar in both genders. Based on our results we suggest a model for gametocyte egress (Fig. 9). The first sign of egress is extension of the membranes and swelling of the erythrocyte. Then the PVM ruptures and vesiculates. This is followed by the opening of a single stabilized pore in the RBCM. The next step had two distinct morphologies in females. In some cases vesicles of the PVM are extruded through the pore followed by vesiculation of the red blood cell membrane. In other cases, the membrane remained in one piece while the gamete gently slid out. After the gamete exit was complete vesiculation of the RBCM immediately took place (Fig. 1g,k, Supplementary Information Videos S2,S3). In male egress we only detected the former type and commonly the extrusion of the vesicles was accompanied by a single flagellum, although in two movies a vesicle was extruded before the flagellum (Fig. 2f,m, Supplementary Information Videos S6,S8). Again, vesiculation of the red blood cell membrane happened rapidly. Interestingly, in one unusual exflagellation event (Supplementary Information Video S9) we detected pore opening happening before the flagella exited the residual cell. This supports the notion that egress of the male is independent of flagellar movement, as has also been described in the *P. berghei* mutant lacking the kinase Pbmap-2^34^. In this mutant male gametocytes egress normally although motile flagella are not formed. Our results from a mutant lacking PPLP2 also showed that although motile flagella were formed they remained trapped within the RBCM^1^.

The swelling of the cell is the first stage of egress. This has also been observed during merozoite egress^30,35,36^, and similar to that we found the PV compartment was extended while the RBC cytoplasm shrunk (Fig. 6). This has been suggested to be the result of water and ion re-distribution between the two comparments^36^. In schizont rupture the swelling takes place several minutes before egress^36^. In our analysis swelling had usually already taken place when recording started. However, in a few events we could time the interval to PVM vesiculation which lasted between approximately 1–6 minutes. This is roughly similar to what has been reported for PVM rupture in *P. falciparum* gametocyte egress^37^. The coincidence of this time interval in schizont and gametocyte egress may point to common processes necessary for the continuation of membrane rupture.

The next step, the rupture and consequent vesiculation of the PVM, was detected in all of the events, suggesting that this is a required step in egress. In schizont rupture the opening of a single stabilized pore in the RBCM, and the outward curling of the membrane results in expulsion of the non-motile merozoites thus assisting in their dispersal^30^. Gamete egress is different; male gametes are highly motile due to the beating of the flagellum and after collision with the non-motile female gametes, fusion of the gametes takes place. Therefore, there is no need for assisted dispersal of these stages. In all cases observed here a single pore was opened in the RBCM, but the subsequent events had two distinct outcomes. On most occasions the opening of the membrane was rapid, and vesiculation occurred quickly following gamete release (Supplementary Information Video S1, S4-S8). In two cases female gametes gently slid out of the RBCM, which for a few seconds remained intact, buckled and then vesiculated (Supplementary Information Videos S2,S3). Although buckling strongly suggests curling, we were not able to clearly detect the characteristic curling of the pore’s rim, which has been described during merozoite and gametocyte egress of *P. falciparum*^30,31^ despite many attempts of visualization of the RBCM using the TER-119 antibody. In some images there is a thickening of the membrane at the pore opening (Figs 3b,c and 5c) but we hesitate to unequivocally state that this is outward curling. The lack of detection of unequivocal curling, however, might be due to the fact that the RBCM destabilizes into vesicles faster than it curls.

After the membrane opening, the RBCM is vesiculated. The time interval between pore opening and RBCM vesiculation varies between a few seconds and several minutes (Fig. S1a). In all cases membrane vesicles were formed. To determine if these vesicles were of the outside-out type, that is the outer leaflet of the RBCM remains outside the vesicle, we used the TER-119 antibody to label the vesicles on fixed but not permeabilized samples at a time point when egress was complete. We detected many labeled vesicles, suggesting that the outer RBCM leaflet is facing outwards (Fig. 4). This observation is not contradictory to curling as the destabilized membrane can re-seal in an outside-out fashion. The lack of a marker for the inner leaflet prevented us from identifying whether any vesicles of inside-out type occur.

In the movies we especially noticed the immediate vesiculation of the RBCM after curling and buckling. In *P. falciparum* merozoite egress vesiculation of the RBCM takes place later^38^. Possibly this difference can be explained by differences in the organization of infected cells and/or the molecular events taking place. The stabilization of the pore and the vesiculation is governed by the state of the cortical cytoskeleton, which is altered in the parasitized red blood cell^39^. RBCM ruptured at one point with the characteristic curling at the pore opening of a *P. falciparum* gametocyte has been described^31^. However, the RBCM seems to remain intact in one piece attached to the emerging gametocyte^2,31,40^, which is different to the vesiculation that we detected here. Less is known about PVM rupture although three studies on *P. falciparum* have revealed similar vesicles to those described here. Immuno-EM using the *Pf*s16 antibody on activated gametocytes detected vesicles heavily labeled with the antibody^5,6^. A TEM study revealed the ruptured PVM first as retracted membranes still attached to the gametocyte and later as flattened vesicles inside the RBCM^2^. As far as we know extrusion of the vesicles has not been reported during *P. falciparum* gametocyte emergence.

Our study confirms that the exit of gametocytes from the host cell involves some differences from that of merozoite egress, suggesting unique aspects of each process. This is underscored by the fact that gametocyte egress involves specific proteins that have no function during merozoite egress (see for example^1,8–10,13,37^). It remains an open question whether gametocyte egress of *P. falciparum* and the rodent parasite are similar. Possibly the differences that we report may be due to different experimental approaches or the fact that the rodent parasite mainly develops in reticulocytes as opposed to the erythrocyte exploited by *P. falciparum*. One advantage of our study is that we followed egress in real time and in live samples, which reduces artefacts that may be introduced by fixation of samples. It is clear that these complex processes must be studied by a combination of methods. Especially important for more detailed future investigations will be defined cellular markers that will allow us to unequivocally follow each membrane and also differentiate the inner and outer leaflet of RBCM and PVM. The fact that gametogenesis is a key transition point in the parasite’s life cycle implies that further studies to understand the cell biology underlying this complex series of events should be of high priority.

## Experimental Procedures

### Ethics statement

All work was carried out in full accordance with Greek regulations consisting of the Presidential Decree (160/91) and law (2015/92) which implement the directive 86/609/EEC from the European Union and the European Convention for the protection of vertebrate animals used for experimental and other scientific purposes and the new legislation Presidential Decree 56/2013. The experiments were carried out in a certified animal facility license (EL91-BIOexp-02) and the protocol has been approved by the FORTH Committee for Evaluation of Animal Procedures (6740/8/10/2014) and by the Prefecture of Crete (license number #27290, 15/12/2014).

### Parasite strains

Parasite strains used in this study were the wild-type ANKA 2.34 and *P. berghei* ANKA 820cl1m1cl1 (RMgm-164), here called 820, in which female gametocytes express red fluorescent protein (RFP), while the males express GFP (Ponzi, 2009). The deletion mutant of *pplp2* and the sop12-GFP have been described^1,33^. The parasite strains were maintained in 6–10 week old Theiler’s original or Swiss mice by intraperitoneal (i.p.) injection of infected blood. Mice were treated with 100 μl of phenylhydrazine (25 mg/ml stock solution) two days prior the infection. Parasitaemia and gametocytemia were determined by Giemsa-stained blood smears.

### Gametocyte activation

Gametocytes were activated after diluting infected blood 1:10 in activation medium (RPMI1640 with L-glutamine, 25 mM Hepes, 2 g/L NaHCO3, 10% FBS, 50 μM XA, pH8.0) supplemented with 50 μM xanthurenic acid and incubating the samples for 10 min at 19 °C.

### Observation protocol

The gametocytes were activated by dilution 1:100 and observation started approximately two min after the activation. A differential interference contrast (DIC) microscope with a 100x objective was used for recording gametocytes. The high-speed imaging was performed using a high-speed camera Phantom V7 (Vision Research Inc). Image analysis was performed using Moviemaker and ImageJ.

### Antibodies

The monoclonal antibody TER-119 conjugated to Alexa Fluor® 488, recognizes a glycophorin A-associated protein on the surface of the red blood cell^41^. The SEP1 antiserum produced in rabbits has been described^42^. GFP was visualized by GFP Tag Monoclonal Antibody from Invitrogen. Secondary antibodies were anti-rabbit (Cy-3, Jackson Immuno-Research) and anti-mouse (Alexa Fluor® 647, Invitrogen).

### Live immunolabeling of the RBCM

An infected blood sample was diluted in activation medium containing the antibody and the samples incubated for 15–20 min at 37 °C. The cells were imaged in an epifluorescence microscope at 19 °C. *P. berghei* ANKA 820cl1m1cl1 (RMgm-164) were used at these experiments.

### Immunofluorescence assays of fixed infected blood samples

The TER-119 antibody was used to label the outer leaflet of the RBCM. Gametocytes were activated and samples were fixed in 4% formaldehyde at different time points after the activation. The samples were not permeabilized. All steps were carried out at room temperature. The parasites were added to poly-L-lysine coated cover slips placed in 24-well plates, centrifuged at 500xg for 15 min, and the fixative removed, followed by washing twice with 1 × PBS. The TER-119 antibody was added diluted 1:200 in PBS with 2% normal goat serum for 1 h incubation. Samples are washed twice with 1 × PBS before mounting in Vectashield (vector laboratories). Image analysis was performed as above.

For immunolabeling of WT and PbSOP12-EGFP parasites with anti-SEP1 antibody non-activated and activated for 8 min samples were permeabilized with 0,1% saponin for 30 sec; at later time points no permeabilization was carried out. Fixation was as described above. Hoechst 33342 was used to stain DNA. For PbSOP12-EGFP parasites anti-GFP antibody was also added.

The *pplp2(−)* mutants were immunolabed with anti- SEP1 as above. Permeabilization was done using equinatoxin.

### Electron microscopy

Samples were fixed in 2.5% glutaraldehyde and processed using the ROTO method^43^ and observed on a FEI Tecnai F30 at 300 kV (Advanced Microscopy Facility, Bio21 Institute, the university of Melbourne, Australia). Micrographs were acquired with a Gatan Ultrascan 1000 2 × 2k. Alternatively the samples were visualized by block face imaging in a Zeiss Sigma equipped with a Gatan 3View system (Center for Microscopy and Microanalysis, University of Queensland, Australia) with a 50 nm section thickness.

## Electronic supplementary material

Supplementary Information

Supplementary Information video S1.

Supplementary Information video S2.

Supplementary Information video S3.

Supplementary Information video S4a.

Supplementary Information video S4b.

Supplementary Information video S4c.

Supplementary Information video S5.

Supplementary Information video S6.

Supplementary Information video S7.

Supplementary Information video S8.

Supplementary Information video S9.

## References

[1] Deligianni E (2013). A perforin-like protein mediates disruption of the erythrocyte membrane during egress of Plasmodium berghei male gametocytes. Cellular microbiology.

[2] Sologub L (2011). Malaria proteases mediate inside-out egress of gametocytes from red blood cells following parasite transmission to the mosquito. Cellular microbiology.

[3] Spielmann T, Montagna GN, Hecht L, Matuschewski K (2012). Molecular make-up of the Plasmodium parasitophorous vacuolar membrane. International journal of medical microbiology: IJMM.

[4] Matthews K (2013). The Plasmodium translocon of exported proteins (PTEX) component thioredoxin-2 is important for maintaining normal blood-stage growth. Molecular microbiology.

[5] Baker DA, Daramola O, McCrossan MV, Harmer J, Targett GA (1994). Subcellular localization of Pfs16, a Plasmodium falciparum gametocyte antigen. Parasitology.

[6] Bruce MC, Carter RN, Nakamura K, Aikawa M, Carter R (1994). Cellular location and temporal expression of the Plasmodium falciparum sexual stage antigen Pfs16. Molecular and biochemical parasitology.

[7] Kongkasuriyachai D, Fujioka H, Kumar N (2004). Functional analysis of Plasmodium falciparum parasitophorous vacuole membrane protein (Pfs16) during gametocytogenesis and gametogenesis by targeted gene disruption. Molecular and biochemical parasitology.

[8] Ponzi M (2009). Egress of Plasmodium berghei gametes from their host erythrocyte is mediated by the MDV-1/PEG3 protein. Cellular microbiology.

[9] Talman AM (2011). PbGEST mediates malaria transmission to both mosquito and vertebrate host. Molecular microbiology.

[10] Deligianni E (2011). Critical role for a stage-specific actin in male exflagellation of the malaria parasite. Cellular microbiology.

[11] Olivieri A (2015). Distinct properties of the egress-related osmiophilic bodies in male and female gametocytes of the rodent malaria parasite Plasmodium berghei. Cellular microbiology.

[12] Rupp I, Bosse R, Schirmeister T, Pradel G (2008). Effect of protease inhibitors on exflagellation in Plasmodium falciparum. Molecular and biochemical parasitology.

[13] Kehrer J, Frischknecht F, Mair GR (2016). Proteomic Analysis of the Plasmodium berghei Gametocyte Egressome and Vesicular bioID of Osmiophilic Body Proteins Identifies Merozoite TRAP-like Protein (MTRAP) as an Essential Factor for Parasite Transmission. Molecular & cellular proteomics: MCP.

[14] Bargieri DY (2016). *Plasmodium Merozoite T*RAP Family Protein Is Essential for Vacuole Membrane Disruption and Gamete Egress from Erythrocytes. Cell host & microbe.

[15] Kafsack BF (2014). A transcriptional switch underlies commitment to sexual development in malaria parasites. Nature.

[16] Sinha A (2014). A cascade of DNA-binding proteins for sexual commitment and development in Plasmodium. Nature.

[17] Silvestrini F (2010). Protein export marks the early phase of gametocytogenesis of the human malaria parasite Plasmodium falciparum. Molecular & cellular proteomics: MCP.

[18] Billker O (1998). Identification of xanthurenic acid as the putative inducer of malaria development in the mosquito. Nature.

[19] McRobert L (2008). Gametogenesis in malaria parasites is mediated by the cGMP-dependent protein kinase. PLoS biology.

[20] Brochet M (2014). Phosphoinositide metabolism links cGMP-dependent protein kinase G to essential Ca(2)(+) signals at key decision points in the life cycle of malaria parasites. PLoS biology.

[21] Raabe AC, Wengelnik K, Billker O, Vial HJ (2011). Multiple roles for Plasmodium berghei phosphoinositide-specific phospholipase C in regulating gametocyte activation and differentiation. Cellular microbiology.

[22] Hale VL (2017). Parasitophorous vacuole poration precedes its rupture and rapid host erythrocyte cytoskeleton collapse in Plasmodium falciparum egress. Proceedings of the National Academy of Sciences of the United States of America.

[23] Taylor HM (2010). The malaria parasite cyclic GMP-dependent protein kinase plays a central role in blood-stage schizogony. Eukaryotic cell.

[24] Chandramohanadas R (2009). Apicomplexan parasites co-opt host calpains to facilitate their escape from infected cells. Science.

[25] Cruz LN (2012). Extracellular ATP triggers proteolysis and cytosolic Ca(2)(+) rise in Plasmodium berghei and Plasmodium yoelii malaria parasites. Malaria journal.

[26] Glushakova S (2013). Cytoplasmic free Ca2+ is essential for multiple steps in malaria parasite egress from infected erythrocytes. Malaria journal.

[27] Blackman MJ, Carruthers VB (2013). Recent insights into apicomplexan parasite egress provide new views to a kill. Current opinion in microbiology.

[28] Millholland MG (2011). The malaria parasite progressively dismantles the host erythrocyte cytoskeleton for efficient egress. Molecular & cellular proteomics: MCP.

[29] Millholland MG (2013). *A host GPCR signaling* network required for the cytolysis of infected cells facilitates release of apicomplexan parasites. Cell host & microbe.

[30] Abkarian M, Massiera G, Berry L, Roques M, Braun-Breton C (2011). A novel mechanism for egress of malarial parasites from red blood cells. Blood.

[31] Suarez-Cortes P, Silvestrini F, Alano P (2014). A fast, non-invasive, quantitative staining protocol provides insights in Plasmodium falciparum gamete egress and in the role of osmiophilic bodies. Malaria journal.

[32] Curra C (2012). Erythrocyte remodeling in Plasmodium berghei infection: the contribution of SEP family members. Traffic.

[33] Sala KA (2015). The Plasmodium berghei sexual stage antigen PSOP12 induces anti-malarial transmission blocking immunity both *in vivo* and *in vitro*. Vaccine.

[34] Tewari R, Dorin D, Moon R, Doerig C, Billker O (2005). An atypical mitogen-activated protein kinase controls cytokinesis and flagellar motility during male gamete formation in a malaria parasite. Molecular microbiology.

[35] Gilson PR, Crabb BS (2009). Morphology and kinetics of the three distinct phases of red blood cell invasion by Plasmodium falciparum merozoites. International journal for parasitology.

[36] Glushakova S (2010). New stages in the program of malaria parasite egress imaged in normal and sickle erythrocytes. Current biology: CB.

[37] Wirth CC (2014). Perforin-like protein PPLP2 permeabilizes the red blood cell membrane during egress of Plasmodium falciparum gametocytes. Cellular microbiology.

[38] Glushakova S, Yin D, Li T, Zimmerberg J (2005). Membrane transformation during malaria parasite release from human red blood cells. Current biology: CB.

[39] Lalle M (2011). Dematin, a component of the erythrocyte membrane skeleton, is internalized by the malaria parasite and associates with Plasmodium 14-3-3. The Journal of biological chemistry.

[40] Eksi S, Williamson KC (2011). Protein targeting to the parasitophorous vacuole membrane of Plasmodium falciparum. Eukaryotic cell.

[41] Kina T (2000). The monoclonal antibody TER-119 recognizes a molecule associated with glycophorin A and specifically marks the late stages of murine erythroid lineage. British journal of haematology.

[42] Birago C (2003). A gene-family encoding small exported proteins is conserved across Plasmodium genus. Molecular and biochemical parasitology.

[43] Hanssen E (2013). Electron tomography of Plasmodium falciparum merozoites reveals core cellular events that underpin erythrocyte invasion. Cellular microbiology.

