# Supplementary material for: Sequential Membrane Rupture and Vesiculation during Plasmodium berghei Gametocyte Egress from the Red Blood Cell
Source: Sci Rep. 2018 Feb 23;8:3543. doi: 10.1038/s41598-018-21801-3 (PMC5824807; doi:10.1038/s41598-018-21801-3)
Supplement: Supplementary file 1 — Supplementary Information [file 41598_2018_21801_MOESM1_ESM.pdf]

Sequential Membrane Rupture and Vesiculation during *Plasmodium berghei* Gametocyte Egress from the Red Blood Cell

Maria Andreadaki[1], Eric Hanssen[3], Elena Deligianni[1], Cyrille Claudet[4], Kai Wengelnik[5], Vanessa Mollard[6], Geoffrey I. McFadden[6], Manouk Abkarian[4], Catherine Braun-Breton[5] and Inga Siden-Kiamos[1*]

[1]Institute of Molecular Biology and Biotechnology, Foundation for Research and Technology - Hellas, Heraklion, Greece

[2]Department of Biology, University of Crete, Heraklion, Greece

[3]Bio21 Molecular Science and Biotechnology Institute, Electron Microscopy Unit and Department of Biochemistry and Molecular Biology, University of Melbourne, Melbourne, Victoria, Australia

[4]Université de Montpellier, CNRS UMR 5048, INSERM UMR 1054, Montpellier, France

[5]Université de Montpellier, CNRS UMR 5235, Montpellier Cedex, France

[6]School of BioSciences, University of Melbourne, Melbourne, Victoria, Australia

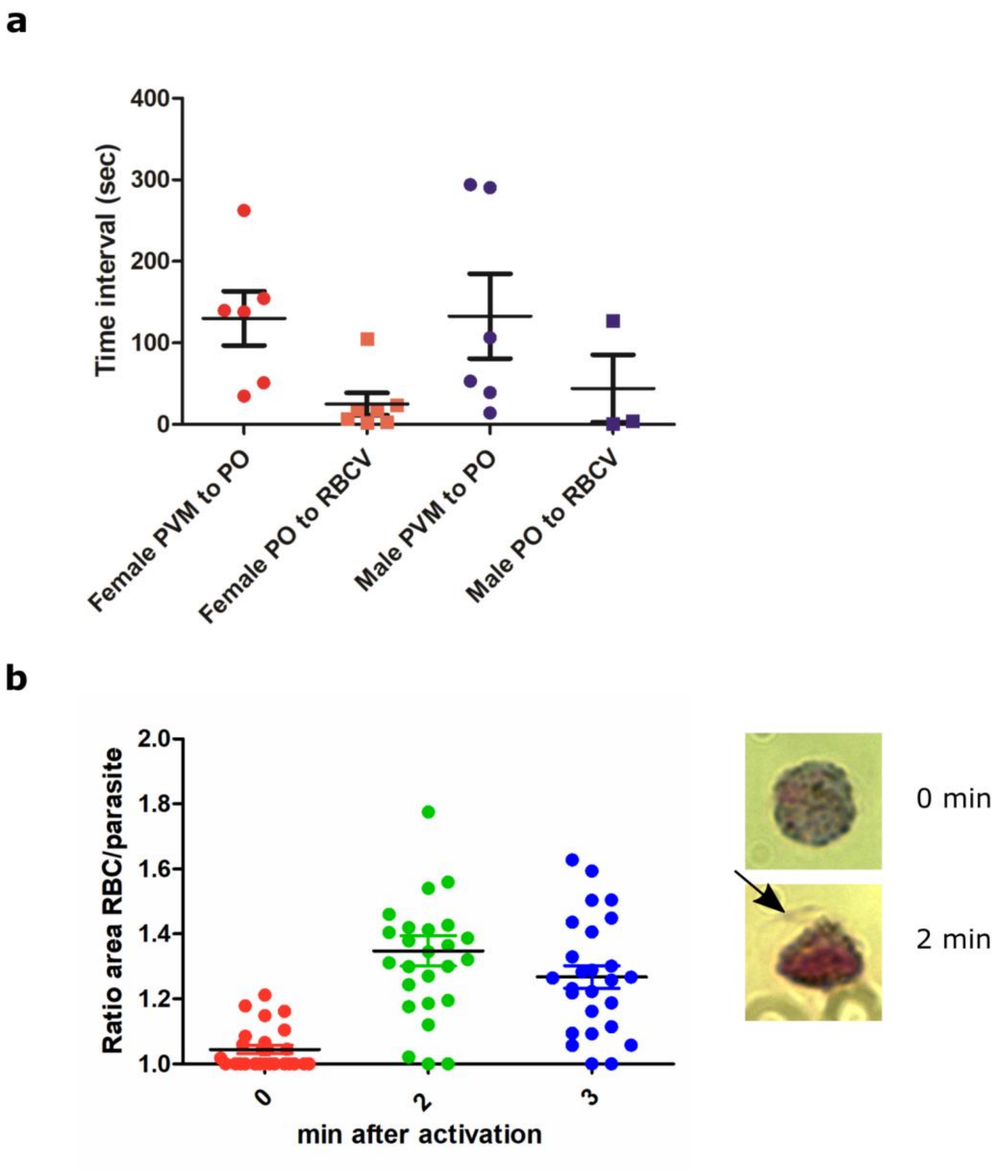

Supplementary Information Fig. S1. a. Time intervals during egress. The time of PVM rupture, pore opening and RBCM vesiculation were determined from movies where these events were clearly visible. Time intervals (sec) are plotted for PVM rupture to pore opening (PVM to PO, circles), pore opening to RBCM vesiculation (PO to RBCV, squares), separately for each gender. Females (red), PVM to PO n=6, PO to RBCV n=7. Males (blue), PVM to PO n=6, PO to RBCV n=3. **b. Swelling of RBCM and PVM.** WT parasites were examined on Giemsa stained smears to quantify swelling of the RBCM and PVM. Infected blood was diluted with RPMI and the samples were either smeared immediately (0) or after incubation at 19°C for 2 or 3 minutes. The areas of the parasite and the host cell were measured and the ratio calculated. 26 parasites for each time point were examined. On the right are two representative images of non-swollen (0 min) and swollen gametocytes. Arrow points to the extended membranes. The differences of the activated cells compared with the 0 min time point are statistically significant (Student's t-test, P<0.0001).

Supplementary Information video S1. Egress of a female gametocyte, corresponding to Female 1 in Fig. 1.
Supplementary Information video S2. Egress of a female gametocyte, corresponding to Female 2 in Fig. 1.
Supplementary Information video S3. Egress of a female gametocyte, corresponding to Female 3 in Fig. 1.
Supplementary Information video S4a. Egress of a female gametocyte, corresponding to Female 4 in Fig. 1. Duration one min, continued in Move S4b.
Supplementary Information video S4b. Continuation of Video S4a. Duration one min, continued in Video S4c.
Supplementary Information video S4c. Continuation of Video S4b with duration 54,82 sec.
Supplementary Information video S5. Egress and exflagellation of male gametocyte, corresponding to Male 1 in Fig. 2.
Supplementary Information video S6. Egress and exflagellation of male gametocyte, corresponding to Male 2 in Fig. 2.
Supplementary Information video S7. Egress and exflagellation of male gametocyte, corresponding to Male 3 in Fig. 2.
Supplementary Information video S8. Egress and exflagellation of male gametocyte, corresponding to Male 4 in Fig. 2.
Supplementary Information video S9. Egress and exflagellation of a male gametocyte. Rupture of the RBCM happens before exit of the flagella from the gametocyte and the flagella. The flagella move slowly suggesting that exflagellation is abnormal.
